# Prevalence and correlates of active syphilis and HIV co-Infection among sexually active persons aged 15–59 years in Zambia: Results from the Zambia Population-based HIV Impact Assessment (ZAMPHIA) 2016

**DOI:** 10.1371/journal.pone.0236501

**Published:** 2020-07-24

**Authors:** Hiwote Solomon, Albertina Ngomah Moraes, Daniel B. Williams, Arlette Simo Fotso, Yen T. Duong, Clement B. Ndongmo, Andrew C. Voetsch, Hetal Patel, Kathryn Lupoli, James B. McAuley, Gina Mulundu, Webster Kasongo, Lloyd Mulenga

**Affiliations:** 1 Doctor of Public Health Program, School of Public Health, Boston University, Boston, MA, United States of America; 2 Ministry of Health Zambia, Ndeke House, Lusaka, Zambia; 3 National Health Research Authority, Lusaka, Zambia; 4 Zambia National Public Health Institute, Lusaka, Zambia; 5 Division of Global HIV & TB, Center for Global Health, Centers for Disease Control and Prevention, Atlanta, Georgia, United States of America; 6 DPS University of the Witwatersrand, Johannesburg, South Africa; 7 ICAP at Columbia University, Mailman School of Public Health, Pretoria, South Africa; 8 ICAP at Columbia University, Mailman School of Public Health, New York, NY, United States of America; 9 USAID Global Health Supply Chain Program, United States Agency for International Development, Arlington, Virginia, United States of America; 10 Rush University Medical Center, Chicago, Illinois, United States of America; 11 University Teaching Hospital, Lusaka, Zambia; 12 School of Medicine, University of Zambia, Lusaka, Zambia; 13 Tropical Disease Research Centre, Ndola, Zambia; University of Alabama at Birmingham, UNITED STATES

## Abstract

**Objectives:**

The main objectives of the study are to estimate HIV prevalence, active syphilis prevalence, and correlates of co-infection with HIV in Zambia, among recently sexually active individuals aged 15 to 59 years old.

**Methods:**

We used data from the 2016 Zambia Population-based HIV Impact Assessment (ZAMPHIA), a national household survey that included biomarker testing for HIV and syphilis. Chembio DPP^®^ Syphilis Screen and Confirm Assay was used to distinguish between active and older syphilis infections. This is the first time Chembio DPP^®^ has been used in a national survey. Log-binominal modelling was utilized to understand the risk of acquiring HIV/active syphilis co-infection using select socio-demographic and sexual behavior variables. Multivariable analysis compared those with co-infection and those with no infection. All reported results account for the complex survey design and are weighted.

**Results:**

A total of 19,114 individuals aged 15–59 years responded to the individual interview and had a valid syphilis and/or HIV test. The prevalence for those sexually active in the 12 months preceding ZAMPHIA 2016 was 3.5% and 13% for active syphilis and HIV, respectively. The prevalence of HIV/active syphilis co-infection was 1.5%. Factors associated with higher prevalence of co-infection versus no infection among females included, but were not limited to, those living in urban areas (adjusted prevalence ratio (aPR) = 3.0, 95% CI = 1.8, 4.8), those had sexual intercourse before age 15 years (aPR = 1.8, 95% CI = 1.1, 2.9), and those who had two or more sexual partners in the 12 months preceding the survey (aPR = 2.7, 95% CI = 1.6, 4.7).

**Conclusion:**

These findings show high prevalence for both mono-infection with HIV and syphilis, as well as co-infection with HIV/active syphilis in Zambia. There is a need for better screening and partner services, particularly among those engaging in high-risk sexual behaviors (e.g., engaging in transactional sex).

## Introduction

Human immunodeficiency virus (HIV) and syphilis affect similar populations, with co-infection between the two groups being common [1–3]. Persons already infected with other sexually transmitted infections (STIs) such as syphilis are 3 to 5 times more likely to acquire HIV if exposed to the virus through sexual contact [4]. This linkage between syphilis and the transmission and acquisition of HIV infection is of major concern as it may temper the gains made in controlling the HIV epidemic, particularly in sub-Saharan Africa [4]. The early detection and treatment of syphilis, therefore, is vital [2]. Additionally, modifications in case management approaches, as well as behavioral changes in response to the HIV epidemic, have resulted in significant changes in the epidemiology of STIs in developing countries [5]. Globally, the past three decades have seen an overall decline in the reported cases of syphilis, with the average prevalence of syphilis in 2015 estimated at 1.11% worldwide; Africa had the highest regional prevalence at 3.04%, while Europe had the lowest at 0.12% [6]. Although sexually transmitted infections such as HIV and syphilis are among the most common reasons for seeking care among adult populations worldwide, they are often undiagnosed and untreated leading to complications and serious consequences beyond the impact of the infection itself, such as stroke, dementia, infertility, and mother-to-child transmission [7–9].

Co-infection of HIV and syphilis often has complex implications and complications relative to mono-infection. For example, HIV infection has been shown to affect the natural history of syphilis and response to treatment, with an increased likelihood of development of neurosyphilis and relapse, with confounded diagnosis most likely through increased incidence of genital ulcers [1,4,7]. Similarly, syphilis has a detrimental impact on HIV infection, resulting in increased viral loads and decreasing CD4 cell counts [3,9].

HIV and syphilis are common infections in Zambia. We conducted an analysis to estimate HIV prevalence, active syphilis prevalence, and correlates of HIV and active syphilis co-infection in Zambia, as part of a nationally representative population-based survey among individuals 15 to 59 years old. Even when data exist through sentinel surveillance, there are data gaps as most data focus on women where the sub-sample size has been smaller than our sub-sample (15–49 years vs. 15–59 years). Additionally, this is the first time the Chembio DPP^®^ Syphilis Screen and Confirm Assay is used in a nationally representative survey.

## Methods

### Study design

Prior data available for syphilis are outdated as the last national prevalence estimate for active syphilis was in the 2007 Zambia Demographic Health Survey (ZDHS). We conducted an analysis using data from the 2016 Zambia Population-based HIV Impact Assessment (ZAMPHIA). ZAMPHIA 2016 was a nationally representative cross-sectional population-based survey of households across all 10 provinces of Zambia. The study used a two-stage stratified sample design. The first stage utilized a probability proportional to size method to select 511 enumeration areas from the 2010 Census of Population and Housing in Zambia. The second stage randomly selected a sample of households in each enumeration area. The eligible survey population included adults and children between the ages of 0 and 59 years who lived in residential households and slept in the eligible household the night before the survey, were willing and able to provide consent or assent in one of the eight survey languages (English, Bemba, Nyanja, Lozi, Tonga, Lunda, Luvale, or Kaonde).

Human subjects and ethical approval for the ZAMPHIA survey was granted by the Zambia National Health Research Ethics Board (Ref: MH/101/23/10-1), the Tropical Diseases Research Centres Ethics Review Committee (Ref: STC/2015/9), and the Institutional Review Boards at the Centres for Disease Control and Prevention (CDC; Atlanta, Georgia, USA) (Ref: #6760). All participants provided written informed consent or assent. Parental or guardian permission and participant assent were required for persons aged below 17 years. Completed household and individual questionnaires and field laboratory data were submitted electronically to a secure cloud server. Laboratory data were cleaned and merged with the final questionnaire database using unique specimen barcodes and study identification numbers. Anonymized data were used for statistical analyses. Sampling weights were computed to adjust for probability of selection, nonresponse, and non-coverage.

### HIV home-based testing and counselling

In accordance with national guidelines, HIV Home-Based Testing and Counselling was conducted in each household. Participants with a non-reactive result on the screening test (Alere Determine™ HIV-1/2 (Abbott Laboratories, Lake Bluff, IL)) were reported as HIV-negative, while those with a reactive screening test result underwent confirmatory testing using Uni-Gold™ HIV-1/2 (Trinity Biotech, Wicklow, Ireland). Those with a reactive screening and confirmatory test were classified as HIV positive. Those that were reactive by Alere Determine™ and non-reactive by UniGold™ HIV-1/2 were asked to return to their preferred health facility for additional testing in 4 weeks. HIV-seropositive participants were referred to HIV care and treatment at a health facility of their choice. For participants under age 17 years, results were provided to a parent or guardian.

### Syphilis testing

The standard algorithm used in the 2007 ZDHS was a rapid plasma reagin (RPR) test for screening and Treponema pallidum hemagglutination (TPHA) for confirmatory testing. For ZAMPHIA, testing for syphilis infection was conducted in each household among participants ages 15–59 years using the Chembio DPP^®^ Syphilis Screen and Confirm Assay (Chembio Diagnostic Systems, Medford, NY) for the simultaneous detection of antibodies against non-Treponemal (N-Trep) and Treponema pallidum (Trep) antigens using whole blood samples. Rapid tests where *both* Trep and N-Trep lines were present (reactive) or where only the Trep or N-Trep line were reactive were followed up with confirmatory testing. Confirmatory testing was conducted using SD Bioline^®^ Syphilis 3.0 Antibody Test (Abbott Laboratories, Lake Bluff, IL) as approved by the Zambian Government. All cases where the control line failed to appear on the Chembio DPP^®^ or SD Bioline^®^ tests were considered invalid and were repeated. If upon repeat the test was still invalid, the result was considered to be indeterminate and the participant was referred for further testing as per the Population-Based HIV Impact Assessment (PHIA) protocol. For ZAMPHIA classification purposes, participants were considered positive for active syphilis if both the Trep and N-Trep lines of the Chembio DPP^®^ Syphilis Screen and Confirm test and the SD Bioline^®^ Trep line test were reactive.

### Variables

Two outcome variables were used in our analysis, active syphilis and HIV/active syphilis co-infection. Active syphilis prevalence was calculated for different sociodemographic and high-risk sexual behavior variables. High-risk sexual behavior was assessed using information captured for respondents who had sexual intercourse in the 12 months preceding the survey (which we define as ‘recently sexually active’). This included a variable indicating whether or not an individual used a condom during their last sexual encounter; the number of partners the respondent had in the last 12 months (one partner vs. two or more partners), the age of first sexual encounter (< 15 years old vs. ≥15 years old), and a variable to measure if the respondent engaged in transactional sex in the past 12 months (answering ‘yes’ or ‘no’ to question “In the last 12-months, have you paid money for sex?”). The multivariable analysis of HIV/active syphilis co-infection controlled for both socio-demographic and high-risk sexual behaviors. The socio-demographic characteristics included marital status, age, urban/rural residence, and education.

### Statistical analyses

This analysis focused on adolescents and adults aged 15 to 59 years. To calculate active syphilis prevalence and prevalence ratios, only those with definitive blood test results and those who had sexual intercourse in the 12 months preceding the survey were included in the sample. The sample used to assess HIV/active syphilis co-infection was limited to those with definitive blood test results and non-missing values for both syphilis and HIV infections, it also excluded those with mono-infection of HIV and of active syphilis. This was done to understand where the greatest need is for treatment among the co-infected and for prevention among the coinfected and not infected population.

Univariate analyses consisted of prevalence calculation of active syphilis infection, and HIV/active syphilis co-infection to show the overall prevalence. Bivariate analyses disaggregated the overall prevalence for each socio-demographic and sexual behavior variable and multivariate analyses utilized log-binominal modeling. The HIV/active syphilis co-infection outcome variable was regressed on all independent variables and the adjusted prevalence ratios and corresponding 95% confident intervals (CI) are presented.

Co-infection analyses for this paper are disaggregated by gender (male vs. female) in order to account for the heterogeneity that can exist between these two groups. All reported results account for the complex survey design and are weighted. The data were analyzed using STATA Version 15.

## Results

Overall, a total of 19,114 individuals aged 15–59 years responded to the individual interview and consented to biomarker testing and had a valid HIV or syphilis test (10,972 were female and 8,142 were male). Of these individuals, 12,510 (65%) individuals (5,092 males and 7,148 females) were sexually active in the 12 months preceding the survey.

### Prevalence of active syphilis infection, and HIV/active syphilis co-infection

**Fig 1:**
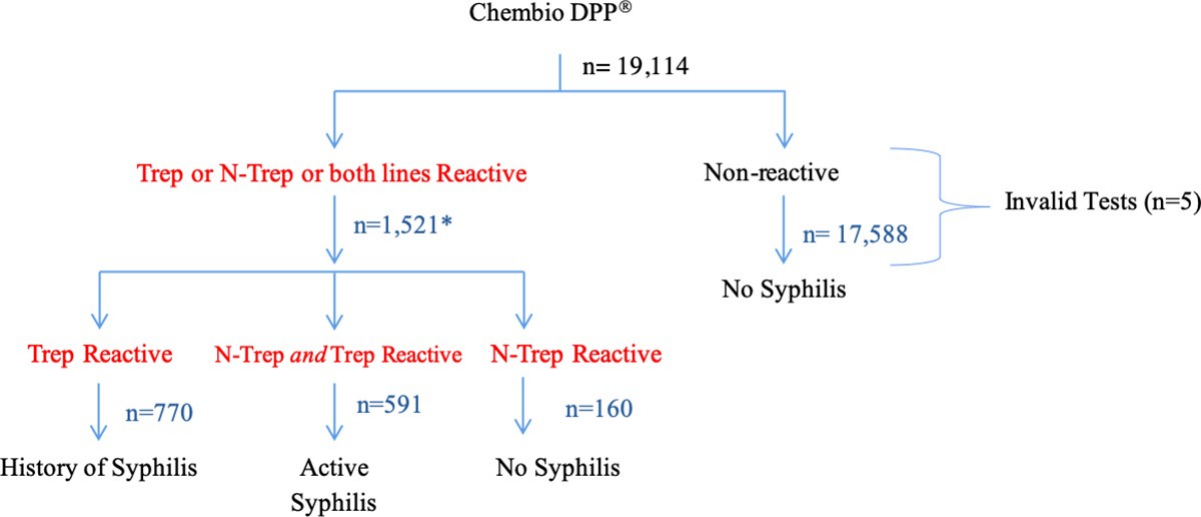
ZAMPHIA 2016 syphilis testing algorithm using Chembio Dual Path Platform (DPP) ^®^ syphilis screen and confirm assay for screening. *1,521 cases were referred for confirmatory testing using SD Bioline^®^.

**Fig 2:**
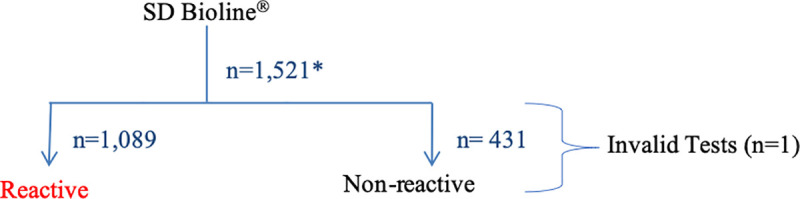
ZAMPHIA 2016 syphilis testing algorithm using SD Bioline^®^ Syphilis 3.0 antibody test for referrals to clinical facilities for treatment.

Of the 19,114 participants tested for syphilis, 1521 had a positive result by Chembio DPP^®^ (Fig 1). Of these, 591 were reactive to both Trep and N-Trep and considered to be positive for active syphilis. Of the 1521 positive results by Chembio DPP^®^, 1089 were reactive on SD Bioline^®^ (Fig 2). Of the 591 cases identified as active syphilis by the Chembio DPP^®^ test, 546 were reactive on SD Bioline^®^ (Table 1).

**Table 1:** ZAMPHIA 2016 syphilis testing results.

		SD Bioline^®^		
		Positive	Negative	Total
Chembio DPP^®^	Trep +	524	246	770
	Non-trep +	19	141	160
	Dual +	546	44	591
	Total	1,089	431	1,521

Table 2 shows the prevalence of active syphilis and HIV infection among select sociodemographic and sexual behavior variables. The national prevalence of active syphilis among those sexually active in the 12 months prior to the survey was 3.5% (95% CI = 3.1, 3.9). Active syphilis prevalence was higher for those aged 25–59 years (3.9% (95% CI = 3.5, 4.5) vs. 2.4% (95% CI = 1.9, 3.1) for 15–24 years old, and those who reported two or more sexual partners (5.3% (95% CI = 4.2, 6.7) vs. 3.2% (95% CI = 2.8, 3.6) for those that reported only one sexual partner.

**Table 2:** Prevalence of active syphilis* and HIV infection among ZAMPHIA 2016 respondents aged 15–59 years who were sexually active in the past 12 months (n = 12,510).

	Active Syphilis Prevalence (95% CI)	HIV Prevalence (95% CI)
Overall	3.47 (3.07–3.92)	12.99 (12.24–13.78)
Sex		
Female	3.93 (3.43–4.50)	14.87 (13.93–15.87)
Male	2.96 (2.51–3.49)	10.88 (10.01–11.81)
Marital Status		
Not in Union	3.83 (3.12–4.69)	12.56 (11.48–13.72)
In Union	3.33 (2.90–3.81)	13.22 (12.29–14.22)
Age		
15–24 years	2.42 (1.89–3.10)	5.10 (4.38–5.93)
25–59 years	3.94 (3.48–4.46)	16.49 (15.44–17.60)
Residence		
Rural	3.18 (2.70–3.74)	9.58 (8.70–10.54)
Urban	3.87 (3.22–4.64)	17.47 (16.22–18.79)
Education		
Less than secondary level	3.89 (3.33–4.54)	11.65 (10.65–12.74)
Secondary level or higher	3.08 (2.60–3.65)	14.26 (13.22–15.36)
Condom use during last sexual encounter in the past 12 months		
No	3.49 (3.07–3.96)	11.19 (10.46–11.97)
Yes	3.36 (2.66–4.24)	20.78 (18.83–22.86)
Number of partners in the past 12 months		
1 sexual partner	3.21 (2.83–3.63)	12.65 (11.88–13.47)
≥ 2 sexual partners	5.30 (4.21–6.66)	15.57 (13.82–17.51)
Age of first sexual encounter		
≥ 15 years	3.49 (3.09–3.94)	13.04 (12.24–13.88)
< 15 years	3.34 (2.46–4.51)	12.52 (10.98–14.25)
Engaged in transactional sex in the past 12 months		
No	3.32 (2.94–3.75)	12.70 (11.94–13.50)
Yes	5.18 (3.40–7.83)	17.37 (14.23–21.04)

*Active syphilis defined as having both Treponemal and Non-Treponemal reactive lines on the Chembio DPP^©^ Syphilis Screen and Confirm Assay (Chembio Diagnostic Systems, Medford, NY) and Trep reactive line on the confirmatory SD Bioline® Syphilis 3.0 Antibody Test

All the estimates are weighted to account for complex survey design.

95% CI = 95% Confidence Interval

The national prevalence of HIV among those sexually active in the last 12 months was 13% (95% CI = 12.2, 13.8). HIV prevalence was higher among females (14.9% (95% CI = 13.9, 15.9)) than among males (10.8% (95% CI = 10.0, 11.8)), those residing in urban areas (17.5% (95% CI = 16.2, 18.8)) compared to those living in rural areas (9.6% (95% CI = 8.7, 10.5)), those between 25 and 59 years (16.7% (95% CI = 15.4, 17.6) compared to those aged 15–24 (5.1% (95% CI = 4.4, 5.9)), those with a secondary level education or higher (14.3% (95% CI = 13.2, 15.4)) vs. those with less than a secondary level of education (11.7% (95% CI = 10.7, 12.7)), those who reported condom use (20.8% (95% CI = 18.8, 22.9)) compared to those who did not use a condom (11.2% (95% CI = 10.3, 12.0)), persons who reported more than one sexual partner (15.6% (95% CI = 13.8, 17.5)) compared to those with one sexual partner (12.7% (95% CI = 11.9, 13.5%)), and those who engaged in transactional sex (17.4% (95% CI = 14.2, 21.0) compared to those who did not (12.7% (95% CI = 11.9, 13.5)).

Among recently sexually active participants aged 15–59 years with a valid HIV or syphilis biomarker test and excluding those with mono-infection (n = 10,683), 1.5% were co-infected with HIV and active syphilis (Table 3). Similar to syphilis mono-infection, co-infection prevalence was statistically higher for respondents aged 25–59 years (2.0% (95% CI = 1.7, 2.4) vs. those aged 15–24 years (0.5% (95% CI = 0.3, 0.8) and those who reported two or more sexual partners (2.8% (95% CI = 1.9, 3.9) vs. 1.3 (95% CI = 1.1, 1.6) for those that reported only one sexual partner. Urban residents also had higher co-infection rates (2.2% (95% CI = 1.8, 2.8) vs 1.0% (95% CI = 0.8, 1.3) for rural residents).

**Table 3:** Multivariable analyses of HIV and active syphilis co-infection by sex among ZAMPHIA 2016 respondents aged 15–59 years who were sexually active in the past 12 months (n = 10,683).

	Overall	Male		Female	
	Prevalence (95% CI)	Prevalence (95% CI)	aPR (95% CI)	Prevalence (95% CI)	aPR (95% CI)
**Overall**	1.49 (1.25–1.77)	1.29 (0.99–1.69)		1.68 (1.35–2.08)	
**Marital Status**					
Not in Union	1.94 (1.47–2.56)	1.03 (0.63–1.70)	1.0	3.19 (2.36–4.29)	1.0
In Union	1.30 (1.05–1.69)	1.42 (1.06–1.88)	0.74 (0.35–1.54)	1.21 (0.92–1.60)	0.33 (0.19–0.56)
**Age**					
15–24 years	0.45 (0.28–0.75)	0.16 (0.04–0.64)	1.0	0.70 (0.41–1.19)	1.0
25–59 years	2.01 (1.67–2.41)	1.81 (1.38–2.37)	14.7 (2.37–91.5)	2.20 (1.75–2.77)	4.38 (2.29–8.38)
**Residence**					
Rural	0.97 (0.76–1.25)	0.98 (0.69–1.40)	1.0	0.97 (0.67–1.38)	1.0
Urban	2.22 (1.75–2.81)	1.73 (1.17–2.56)	1.49 (0.76–2.90)	2.67 (2.06–3.46)	2.95 (1.82–4.79)
**Education**					
Less than secondary level	1.50 (1.19–1.88)	0.97 (0.63–1.48)	1.0	1.85 (1.44–2.39)	1.0
Secondary level or higher	1.48 (1.16–1.89)	1.51 (1.09–2.11)	1.28 (0.63–2.62)	1.44 (1.06–1.96)	0.56 (0.36–0.87)
**Condom use during last sexual encounter in the past 12 months**					
No	1.38 (1.13–1.67)	1.18 (0.88–1.59)	1.0	1.54 (1.20–1.98)	1.0
Yes	2.05 (1.44–2.92)	1.63 (0.94–2.81)	1.34 (0.56–3.18)	2.70 (1.80–4.02)	1.32 (0.76–2.28)
**Number of partners in the past 12 months**					
1 sexual partner	1.33 (1.10–1.60)	1.14 (0.84–1.53)	1.0	1.48 (1.19–1.84)	1.0
≥2 sexual partners	2.75 (1.94–3.89)	1.91 (1.17–3.09)	1.38 (0.76–2.50)	8.21 (5.12–12.9)	2.74 (1.60–4.71)
**Age of first sexual encounter**					
≥15 years	1.49 (1.23–1.78)	1.37 (1.01–1.84)	1.0	1.60 (1.27–2.01)	1.0
< 15 years	1.54 (1.00–2.35)	0.92 (0.44–1.93)	0.91 (0.39–2.12)	2.39 (1.51–3.77)	1.76 (1.09–2.86)
**Engaged in transactional sex in the past 12 months**					
No	1.40 (1.17–1.67)	1.16 (0.87–1.53)	1.0	1.61 (1.30–1.99)	1.0
Yes	3.52 (2.03–6.04)	2.68 (1.35–5.23)	2.32 (1.10–4.87)	9.41 (3.96–20.7)	2.80 (1.40–5.58)
**Pregnancy Status**					
Not currently pregnant				1.76 (1.41–2.20)	1.0
Currently pregnant				0.76 (0.31–1.82)	0.68 (0.28–1.60)

aPR: Adjusted Prevalence Ratio; 95% CI: 95% Confidence Interval. All the estimations are weighted using PHIA biomarker weights.

### Factors associated with HIV/active syphilis co-infection

Multivariable analysis results are reported in Table 3. Adjusted prevalence ratios are shown independently for males and females. For both males and females, the adjusted prevalence ratio of HIV/active syphilis co-infection was significantly higher for those aged 25–59 years (vs. 15–24 years) (aPR_males_ = 14.7 (95% CI = 2.4, 91.5); aPR_females_ = 4.4 (95% CI = 2.3, 8.4)) and persons who engaged in transactional sex (aPR_males_ = 2.3 (95% CI = 1.1, 4.9); aPR_females_ = 2.8 (95% CI = 1.4, 5.6)). For females, additional statistically significant risk factors for HIV/active syphilis co-infection included urban residence (adjusted prevalence ratio (aPR) = 3.0 (95% CI = 1.8, 4.8) vs. rural residence; having had two or more sexual partners (aPR = 2.7 (95% CI = 1.6, 4.7)) vs. only one sexual partner; and early sexual debut (aged <15 years at first sexual encounter) (aPR = 1.8 (95% CI = 1.1, 2.9) vs. aged >15 years at first sexual encounter. Females who reported being married or living with a partner (“in union”), and having a secondary or higher level of education, had lower statistically significant adjusted prevalence ratios (aPR = 0.33 (95% CI = 0.19, 0.56) and aPR = 0.56 (95% CI = 0.36, 0.87), respectively).

## Discussion

ZAMPHIA 2016 revealed the current state of the HIV and syphilis problem in Zambia. Overall, 3% of adolescents and adults 15–59 years were infected with active syphilis, and 12% were infected with HIV [10]. These numbers were slightly higher for those who were sexually active in the 12 months preceding ZAMPHIA 2016 survey (3.5% for syphilis and 13% for HIV). Overall co-infection prevalence for those recently sexually active persons who were co-infected with HIV and active syphilis was 1.3% [10]. The active syphilis prevalence of 3.5% in this study was lower than in previous surveys in Zambia where the prevalence was found to be 7% in the 2001–2002 ZDHS and 4.2% in the 2007 ZDHS [11,12]. The decline in syphilis prevalence over time is not unique to Zambia; similar trends have been observed in several other countries across Eastern and Southern Africa, where a regression analysis indicated a linear relationship between HIV and syphilis incidence that concurrently switched to a negative direction over time during the HIV epidemic [13,14]. However, the 3.5% prevalence in this survey among sexually active persons remains higher compared to recent measures of active syphilis in other countries such Rwanda, where the active syphilis prevalence was found to be 0.9%, and 1.8% in both Kenya and Uganda [15]. Additionally, the Zimbabwe PHIA (2015–2016) found the active syphilis prevalence among persons aged 15–64 years was only 0.8% [16].

ZAMPHIA 2016 is the first nationally representative survey to use the Chembio DPP^®^ test to identify cases of syphilis. For validation purposes, a prior evaluation was conducted at the University of Zambia Virology Lab using the standard minimum of 2,000 specimens comparing Chembio DPP^®^ to SD Bioline^®^ at 99% sensitivity and 99% specificity. Our analysis found that of the 591 cases identified as active syphilis by the Chembio DPP^®^ test, 44 of these cases were non-reactive on SD Bioline^®^. This means that if SD Bioline^®^ is still the approved screening tool, this may provide evidence that around 7% of active syphilis cases may be missed and not referred for treatment. Thus, more appropriate methods of screening and confirming cases of syphilis should be prioritized to avoid over- or under-treating.

As compared to previous surveys done in Zambia the syphilis and HIV prevalence were consistently higher among recently sexually active women in this survey compared to men (3.9% vs 3.0% for syphilis, and 14.9% and 10.9% for HIV, respectively) [11]. In a retrospective analysis among a cohort of HIV discordant couples in Zambia and Rwanda found that syphilis prevalence was higher among women than men (20% vs. 15%) in Zambia [17]. Among the prevalent cases in this analysis, 35% of syphilis positive participants had syphilis positive spouses; 27% of positive women had syphilis positive husbands, whereas 45% of syphilis positive men had syphilis positive wives [17]. Factors that could contribute to higher risks for STIs for women include older sexual partners, earlier marriages, and limited ability to negotiate sexual relationships [15]. High rates of active syphilis among women are of particular concern because infants born to women with syphilis are at a higher risk for congenital syphilis [18,19]. Additionally, high prevalence of HIV increases chances for mother-to-child transmission of HIV infection [14]. The significant difference in the prevalence of active syphilis among our two age groups (15–24 years and 25–59 years) were consistent with other studies conducted in Rwanda and Uganda [15,16, 20].

Among those with recent sexual activity, the co-infection prevalence was 2 times higher among those with more than one sexual partner in the previous year. The use of a condom in the last sexual encounter was associated with a higher co-infection prevalence as compared to those who did not use a condom; a similar finding was seen in a study in Kenya where consistent condom use with the last sex partner was associated with higher HIV prevalence [21]. Previous literature has noted this could be because those aware of their status are more likely to use condoms [21].

Limitations of the study include the inherent bias that is common in cross-sectional surveys, as data will always reflect determinants of survival and/or etiology thus nonparticipants may have been excluded due to factors common with HIV infection and syphilis (hospitalization or death), which may result in the underestimation of the true burden of both active syphilis and co-infection with HIV in the country [22]. The information gathered on sexual risk behavior in the year prior to the survey may be subject to recall bias; some participants may not have felt comfortable talking about their sexual behavior. Our sample for the multivariable analysis was restricted to the recently sexually active who had no infection or those who were co-infected with HIV and active syphilis. While this was done to show the rates of co-infection and compare it with a “positive” situation of no infection in the population, there is a need to assess mono-infection vs. co-infection of HIV and active syphilis.

There is need for further understanding of the epidemiology of HIV infection and STIs in order to assess the potential for the spread of HIV/STIs within sexual networks and develop innovative public health strategies to control new and resurgent epidemics. The reciprocal effect of syphilis and HIV on transmission and disease progression stresses the need for close serologic examination and continuation of care including scaling up of syphilis screening and treatment, particularly among HIV-infected adults [4]. For instance, Zambia and Uganda introduced rapid syphilis testing into prevention of mother-to-child transmission of HIV services and effectively increased screening and treatment for syphilis among HIV positive pregnant mothers, without compromising the HIV services [23]. However, presently routine syphilis antibody testing and treatment is only offered in Zambia during antenatal care visits. Hence, up to date and population-based data are needed. A population-based estimate of syphilis antibody prevalence through this survey should allow Zambia to focus enhanced syphilis antibody testing and treatment to demographics with the highest prevalence, including sexually active women aged 25–59 who engage in high-risk sexual behaviors. Other strategies include syphilis and HIV prevention programs that target populations most at risk for co-infection, and the use of an HIV/Syphilis dual test for improved HIV and syphilis screening, as demonstrated in a six country laboratory evaluation of the rapid diagnostic test [24, 25]. Screening and sexual health education programs about sexually transmitted infections in Zambia should aim to target women of reproductive age.
